# The calcineurin–NFATc pathway modulates the lipid mediators in BAL fluid extracellular vesicles, thereby regulating microvascular endothelial cell barrier function

**DOI:** 10.3389/fphys.2024.1378565

**Published:** 2024-05-15

**Authors:** Manjula Karpurapu, Yunjuan Nie, Sangwoon Chung, Jiasheng Yan, Patrick Dougherty, Sonal Pannu, Jon Wisler, Ryan Harkless, Narasimham Parinandi, Evgeny Berdyshev, Dehua Pei, John W. Christman

**Affiliations:** ^1^ Division of Pulmonary, Critical Care and Sleep Medicine, Ohio State University Wexner Medical Center, Davis Heart and Lung Research Institute, Columbus, OH, United States; ^2^ Department of Basic Medicine, Wuxi School of Medicine, Jiangnan University, Wuxi, Jiangsu, China; ^3^ Department of Pharmacology, Ohio State University, Columbus, OH, United States; ^4^ Department of Chemistry and Biochemistry, Ohio State University, Columbus, OH, United States; ^5^ Department of Surgery, Ohio State Wexner Medical Center, Columbus, OH, United States; ^6^ Division of Pulmonary Critical Care and Sleep Medicine, National Jewish Health, Denver, CO, United States

**Keywords:** acute lung injury, endothelial permeability, extracellular vesicles, eicosanoids, AA metabolome

## Abstract

Extracellular vesicles mediate intercellular communication by transporting biologically active macromolecules. Our prior studies have demonstrated that the nuclear factor of activated T cell cytoplasmic member 3 (NFATc3) is activated in mouse pulmonary macrophages in response to lipopolysaccharide (LPS). Inhibition of NFATc3 activation by a novel cell-permeable calcineurin peptide inhibitor CNI103 mitigated the development of acute lung injury (ALI) in LPS-treated mice. Although pro-inflammatory lipid mediators are known contributors to lung inflammation and injury, it remains unclear whether the calcineurin-NFATc pathway regulates extracellular vesicle (EV) lipid content and if this content contributes to ALI pathogenesis. In this study, EVs from mouse bronchoalveolar lavage fluid (BALF) were analyzed for their lipid mediators by liquid chromatography in conjunction with mass spectrometry (LC-MS/MS). Our data demonstrate that EVs from LPS-treated mice contained significantly higher levels of arachidonic acid (AA) metabolites, which were found in low levels by prior treatment with CNI103. The catalytic activity of lung tissue cytoplasmic phospholipase A2 (cPLA2) increased during ALI, correlating with an increased amount of arachidonic acid (AA) in the EVs. Furthermore, ALI is associated with increased expression of cPLA2, cyclooxygenase 2 (COX2), and lipoxygenases (5-LOX, 12-LOX, and 15-LOX) in lung tissue, and pretreatment with CNI103 inhibited the catalytic activity of cPLA2 and the expression of cPLA2, COX, and LOX transcripts. Furthermore, co-culture of mouse pulmonary microvascular endothelial cell (PMVEC) monolayer and NFAT-luciferase reporter macrophages with BALF EVs from LPS-treated mice increased the pulmonary microvascular endothelial cell (PMVEC) monolayer barrier permeability and luciferase activity in macrophages. However, EVs from CNI103-treated mice had no negative impact on PMVEC monolayer barrier integrity. In summary, BALF EVs from LPS-treated mice carry biologically active NFATc-dependent, AA-derived lipids that play a role in regulating PMVEC monolayer barrier function.

## Graphical Abstract

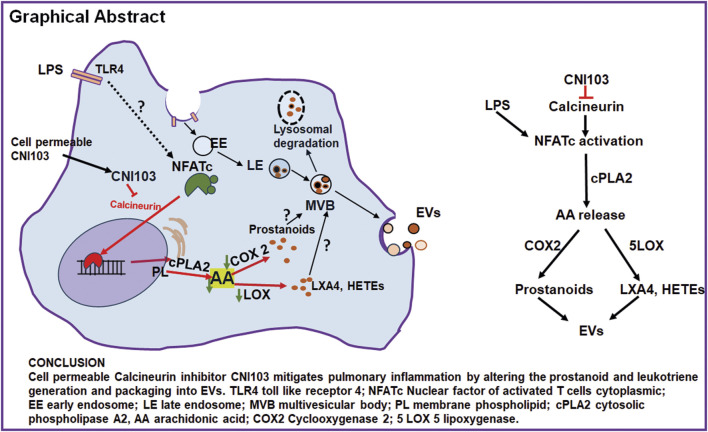

## Introduction

Acute respiratory distress syndrome (ARDS) is characterized by a sustained inflammatory cascade that fails to resolve in time, leading to pulmonary tissue injury and respiratory failure. Lipid mediators derived from ω-6 and ω-3 polyunsaturated fatty acids (PUFA) play a pivotal role in both the initiation and resolution of inflammation. Lipid mediators offer insights into the inflammatory status of ARDS patients (Cioccari et al., 2020). ARDS causes an increased release of arachidonic acid (AA) from the cell membrane phospholipids by the cytosolic phospholipase A2 (PLA2) (Nagase et al., 2000; Nagase et al., 2003; Nagase, 2013). Different classes of cPLA2 are implicated in the pathophysiology of ARDS, and this enzyme is a major source of free AA generation within cells (Kitsiouli et al., 2009). Cellular AA is metabolized by three different enzymatic pathways to generate a wide array of 20 carbon-containing lipid mediators, collectively named eicosanoids (Hanna and Hafez, 2018). AA is metabolized by three different catalytic pathways. The first pathway includes cyclooxygenase (COX 1 and COX2) to generate prostanoids (PGE2, PGD2, and PGF2α), prostacyclin (PGI2), and thromboxanes (TXA2). The second lipoxygenase (LOX) pathway is catalyzed by 5-LOX, 8-LOX, 12-LOX, and 15-LOX (12/15-LOX in mice), producing leukotrienes (LTA4, LTB4, LTC4, LTD4, and LTE4), lipoxins (LXA4 and LXB4), and 8-, 12-, or 15-hydroperoxyeicosatetraenoic acid (HPETE) compounds. Alternatively, metabolic conversion of AA by the CYP450 pathway (CYP450 epoxygenase and CYP450 ω-hydroxylase) generates epoxyeicosatrienoic acid (EETs) and hydroxyeicosatetraenoic acid compounds (HETEs). Eicosanoids are synthesized in response to diverse stimuli, can act as cell signaling molecules at significantly low concentrations, and play a critical role in both normal and disease physiology through paracrine or autocrine actions (Meng et al., 2023; Das, 2021; Aso et al., 2012; Birukova et al., 2007; von Moltke et al., 2012; Yamaguchi et al., 2022).

Extracellular vesicles (EVs) are lipid bilayer membrane-bound vesicles released extracellularly and originate from the cellular endosomal system (small EVs) or plasma membrane (microvesicles, or MVs) (Hessvik and Llorente, 2018; Russell et al., 2019; Vidal, 2019). EVs transfer bioactive cargo, including nucleic acids, lipids, peptides, and other membrane protein receptors, to the recipient cells (Lee et al., 2016; Haggadone and Peters-Golden, 2018; Zhang X. F. et al., 2019; Zhang D. et al., 2019; Yuan et al., 2019). Studies indicate that EV-transported cargo is stable because the EV’s bilayer lipid membranes are enriched in saturated lipids compared to their parent cells (Record et al., 2014; Skotland et al., 2019; Skotland et al., 2020). The applications of EVs as therapeutic targets, biomarkers, novel drug delivery agents, and standalone therapeutics are being actively explored (Lee et al., 2018; Popowski et al., 2020). However, the study of EVs is still in its nascent stages concerning ARDS. A handful of studies implicate the possible role of EV-carried micro-RNAs (miRs) and proteins in regulating inflammatory signaling pathways in different pulmonary diseases, including ARDS (Matthay and Abman, 2018; Soni et al., 2019; Mohan et al., 2020; Wisler et al., 2022).

In our recent study, we demonstrated the functional role of lipid mediators packaged in BALF EVs from ALI mice (Nirujogi et al., 2022). We have observed that BALF EVs from lipopolysaccharide- (LPS)-treated mice could elicit barrier dysfunction in alveolar epithelial cell (AEC) monolayers. However, current research suggests that lipid mediators are short-lived biomolecules that act close to the site of synthesis in circulation or pulmonary edema fluid after their generation (von Moltke et al., 2012; Matthay et al., 1984; Ratnoff et al., 1988). In contrast, studies from our group and others demonstrate that lipids transported by small EVs are stable by virtue of their saturated lipid-rich bilayer membrane. Furthermore, during inflammatory conditions, the disrupted intercellular barrier increases the reach of EVs to distant cells (Zhang et al., 2021; Soni et al., 2022; Tan et al., 2023). In this context, our study investigates the important question of whether EVs released into BALF during ARDS modulate the function of the lung microvascular endothelial cells, resulting in pulmonary edema.

Calcineurin, a calcium-dependent phosphatase, is shown to regulate inflammatory signaling pathways in immune cells (Fric et al., 2012; Zanoni and Granucci, 2012). Calcineurin is a key player in the signaling cascade activated by increased intracellular calcium levels and is well-known for its involvement in T-cell activation and the subsequent immune response (Vaeth and Feske, 2018). Of the several downstream targets of calcineurin is the nuclear factor of activated T-cells (NFAT), which includes four members (NFATc1–NFATc4) (Zanoni and Granucci, 2012; Pan et al., 2013). Upon activation, calcineurin dephosphorylates NFAT, enabling its translocation into the nucleus and subsequent regulation of target gene expression. The genes targeted by NFAT include those involved in cytokine production and inflammatory responses (Vaeth and Feske, 2018). Furthermore, NFAT regulates the expression of COX2 that generates PGE2 from AA (Bhattacharjee et al., 2016).

Our previous studies demonstrate that NFATc3 is activated in pulmonary macrophages in mice subjected to LPS or polymicrobial abdominal sepsis by cecal ligation and puncture (Ranjan et al., 2014; Karpurapu et al., 2018). NFATc3 is shown to regulate a wide array of genes, including cytokines, chemokines, induced nitric oxide synthase, and toll-like receptor 9 (Karpurapu et al., 2018). We developed a cell-permeable peptidyl inhibitor of calcineurin CNI103 that inhibits the activation of NFATc3 by calcineurin. CNI103 significantly decreases the intensity of LPS-induced ALI in mice when administered by intranasal or systemic routes (Dougherty et al., 2020). In the present manuscript, we describe how CNI103 modifies the lipid mediators of BALF EVs and elucidate the functional differences in these EVs that regulate lung endothelial permeability.

## Materials and methods

### Mouse models of LPS-induced ALI

C57BL/6 mice (Stock no. 000664) were purchased from the Jackson Research Laboratories (Bar Harbor, ME, United States) and maintained in the pathogen-free vivarium at OSU DHLRI. All the experiments were conducted on 8–12-week-old male mice, following protocols approved by the Institutional Animal Care and Use Committee of The Ohio State University, protocol # 2013A00000105-R3. Control (VAVAA) or calcineurin inhibitor (CNI103) peptides were delivered via the intranasal route (4 mg/kg in 25 µL 0.01% DMSO) into anesthetized mice. After 2 h, these two groups of mice were subsequently treated with *Escherichia coli* LPS Serotype O55:B5 S-form (4 mg/kg in 25-μL saline, intranasal route). Control mice received an equal volume of saline. After 3 days of LPS treatment, mice from different experimental groups were euthanized with ketamine and xylazine and used for BALF collection. BALF was further used for MV and EV isolation, and the MV/EV-depleted supernatant was used for TNF-α, IL 6, and extravasated protein measurements. Cytokines were assayed using R&D Systems ELISA kits TNF-α (# MTA00B) and IL-6 (#M6000B). In parallel experimental groups of mice, lung tissue was collected directly into Direct-Zol for RNA isolation.

### Isolation of EVs

BALF EVs of 100–180 nm size were isolated by ultracentrifugation as described previously (Nirujogi et al., 2022). In brief, BALF from each mouse was collected by aspiration after instilling 1 mL of PBS three times. Cells and MV-depleted BALF were filtered through 0.22-μM syringe filters and centrifuged using a TLA100 fixed angle rotor on a Beckman Coulter ultracentrifuge at 100,000 g for 6 h at 4°C. The EV pellet was washed in 1 mL PBS and centrifuged at 100,000 g before final reconstitution in PBS for downstream analyses.

#### EV size determination and transmission electron microscopy (TEM)

The size of BALF EVs was measured by the NanoSight nanoparticle tracking analyzer at the Flow Cytometry Facility of the OSU Comprehensive Cancer Center. Comparative evaluations of mean particle size and numbers were conducted across different experimental mice. Photomicrographs of the EVs were obtained through negative staining utilizing the FEI Tecnai G2 Biotwin transmission electron microscope at the OSU Comprehensive Cancer Center-Campus Microscopy and Instrumentation facility.

### Analysis of EV lipid mediators

Lipids were extracted by a modified Bligh and Dyer approach with 0.1 N HCl to induce phase separation and improve free fatty acid and eicosanoid recovery into a chloroform layer (Bligh and Dyer, 1959). Liquid chromatography-electrospray ionization-tandem mass spectrometry (LC-ESI-MS/MS) was performed using a Sciex 6500 QTRAP triple–quadrupole ion trap hybrid mass spectrometer interfaced with a Shimadzu Nexera-X2 UHPLC system. Free fatty acids and their oxidized derivatives were separated on the Ascentis Express C18 column (2.1 × 50 mm, 2.7 μm particle size). Chromatography was performed using gradient elution from solvent A (methanol: water: formic acid 30:70:0.1) to solvent B (methanol with 0.1% formic acid) at 0.65 mL/min flow rate. Deuterated arachidonic, eicosapentaenoic, and docosahexaenoic fatty acids and deuterated prostaglandins, leukotrienes, and iso-prostanes (all from Cayman Chemicals, Ann Arbor, MI) were added during initial steps of extraction at 100 ng/sample (free fatty acids) or 10 ng/sample (prostaglandins, leukotrienes, and isoprostanes) to ensure molecule quantitation through the isotope dilution approach. Parameters of declustering potential and collision energy for each molecule were optimized during infusion experiments.

#### Quantitative real-time PCR (QRT-PCR)

Total RNA from the lung tissue was isolated using a Direct-zol RNA Kit as per the manufacturer’s instruction (Zymo Research, Irvine, CA). The kit includes DNase to remove contaminating genomic DNA from the total RNA, from which cDNA was synthesized, using a RevertAid First-Strand cDNA Synthesis Kit (Thermofisher Scientific, Waltham, MA). Gene expression was measured by quantitative PCR on a Roche LightCycler 480. Target gene expression levels were quantified using the 2 (^−∆∆^Ct) method normalized to GAPDH expression. The different target gene primers used for qPCR are listed in Table 1.

**TABLE 1:** List of primer sequences used in the study.

S. No	Primer	5′- 3′ sequence
1	cPLA2	Forward 5′- GAT GAG GCT CAA GGA CCC AAA G - 3′ Primer 5′- GAA TAA AGC CGA GTC GCT CAC C - 3′
2	COX1	Forward 5′- GAA TGC CAC CTT CAT CCG AGA AG - 3′ Reverse 5′- GCT CAC ATT GGA GAA GGA CTC C - 3′
3	COX2	Forward 5′- GCG ACA TCA TCA AGC AGG AGC - 3′ Reverse 5′- AGT GGT AAC CGC TCA GGT GTT - 3′
4	5-LOX	Forward 5′- GCA GCC ATT CAG GAA CTG GTA - 3′ Reverse 5′- TCT TCC TGG CAC GAC TTT GCT - 3′
5	12-LOX	Forward 5′- CTC TTG TCA TGC TGA GGA TGG AC - 3′ Reverse 5′- AAG AGC CAG GCA AGT GGA GGA T - 3′
6	15-LOX	Forward 5′- GAC ACT TGG TGG CTG AGG TCT T - 3′ Reverse 5′- TCT CTG AGA TCA GGT CGC TCC T - 3′
7	GAPDH	Forward 5′- TGG AAC AAG GAG GAG CAG AGA GCA - 3′ Reverse 5′- TAC TCG CGG CTT TAC GGG - 3′

##### cPLA2 activity assay

The enzymatic activity of cPLA2 in lung tissue was determined by assaying arachidonoyl thio-PC as the substrate, provided in the cPLA2 assay kit, following the manufacturer’s instructions (Cayman Chemical, Ann Arbor, MI, United States). The specific activity was expressed as μmol of arachidonoyl thio-PC hydrolyzed per minute per milligram of total lung cellular protein.

### Analysis of EV uptake

Primary mouse PMVECs were purchased from Cell Biologics (Chicago, IL, United States) and cultured in endothelial cell growth medium with VEGF and other growth factors, penicillin and streptomycin, and EV-depleted FBS. EVs were labeled with PKH26 and purified using density gradient ultracentrifugation on a sucrose gradient, as per the instructions provided by the manufacturer (Sigma-Aldrich, St. Louis, MO, United States). PKH26-labeled EVs (1 × 10^9^/well) were co-cultured with mouse PMVECs (0.04 × 10^6^ cells/well) in black clear bottom 96-well plates for 16 h. Similarly, PKH26-labeled EVs were added to NFAT-luciferase reporter macrophages (1 × 10^9^ EVs/0.04 × 10^6^ cells) grown on 96-well plates and co-cultured for an additional 16 h.

Following incubation with PKH26-labeled EVs, both PMVECs and NFAT-luc cells were gently washed with 1× HBSS and incubated with CD9-Exo-Flow capture beads (System Biosciences, Palo Alto, CA, United States) for 1 h to facilitate adsorption of EVs that were not internalized but were loosely attached to cell membranes. CD9-capture beads were removed, cells were washed with 1× HBSS, and the relative internalization of EVs was quantified using a fluorescence plate reader (excitation/emission at 530 nm and 567 nm). Internalization of EVs was expressed as relative fluorescence units (RFU). Baseline fluorescence from cells incubated in the presence of unlabeled EVs was included for normalization.

### NFAT-luciferase reporter cell activity

NFAT-luciferase RAW 264.7 cells (Abeomics, San Diego, CA) were grown in 96-well plates containing DMEM supplemented with 10% FBS, 1% penicillin, streptomycin, and 3 μg/mL puromycin. These cells express the Renilla luciferase reporter gene under the transcriptional control of the NFAT response element. After stimulation with 100 ng/mL LPS or co-culture with BALF EVs, cells were washed and lysed in 1X cell lysis buffer. The relative transcriptional activation of NFAT in 20 µL of cell lysates was assayed using the Renilla luciferin substrate as per the manufacturer’s instructions.

### PMVEC barrier function

PMVEC monolayer barrier integrity was determined as described previously (Karpurapu et al., 2018). Mouse primary PMVECs (Cell Biologics, Chicago, IL) were grown to 100% confluence (0.2 × 10^6^) on collagen-coated transwells (24-well plates) and co-cultured with 5 × 10^9^ EVs from different groups of mice for an additional 24 h. A 100-μg/mL aliquot of fluoro isothiocyanate (FITC) dextran was added to the transwells, and the flux of FITC dextran into bottom wells was measured using 100 μL medium from the basolateral chamber after 1 h of FITC addition. An equal volume of the culture medium was added to the chamber to compensate for the reduction in volume due to sampling. Fluorescence was measured using a plate reader at 492 nm/520 nm excitation/emission.

### Statistical analysis

All data are expressed as mean ± SEM, n = 5 in each group in the *in vitro* experiments, repeated three times. Representative data sets were presented. In the *in vivo* mouse experiments, n = 10 in the LPS groups, and n = 6–8 in the saline groups. Statistical analyses were performed using GraphPad Prism Version 10.0. The differences between the four experimental groups were determined using ANOVA with a *post hoc* Bonferroni correction test. A *p*-value < 0.05 was considered significant.

## Results

### NFATc regulates the packaging of lipid mediators into EVs

**FIGURE 1:**
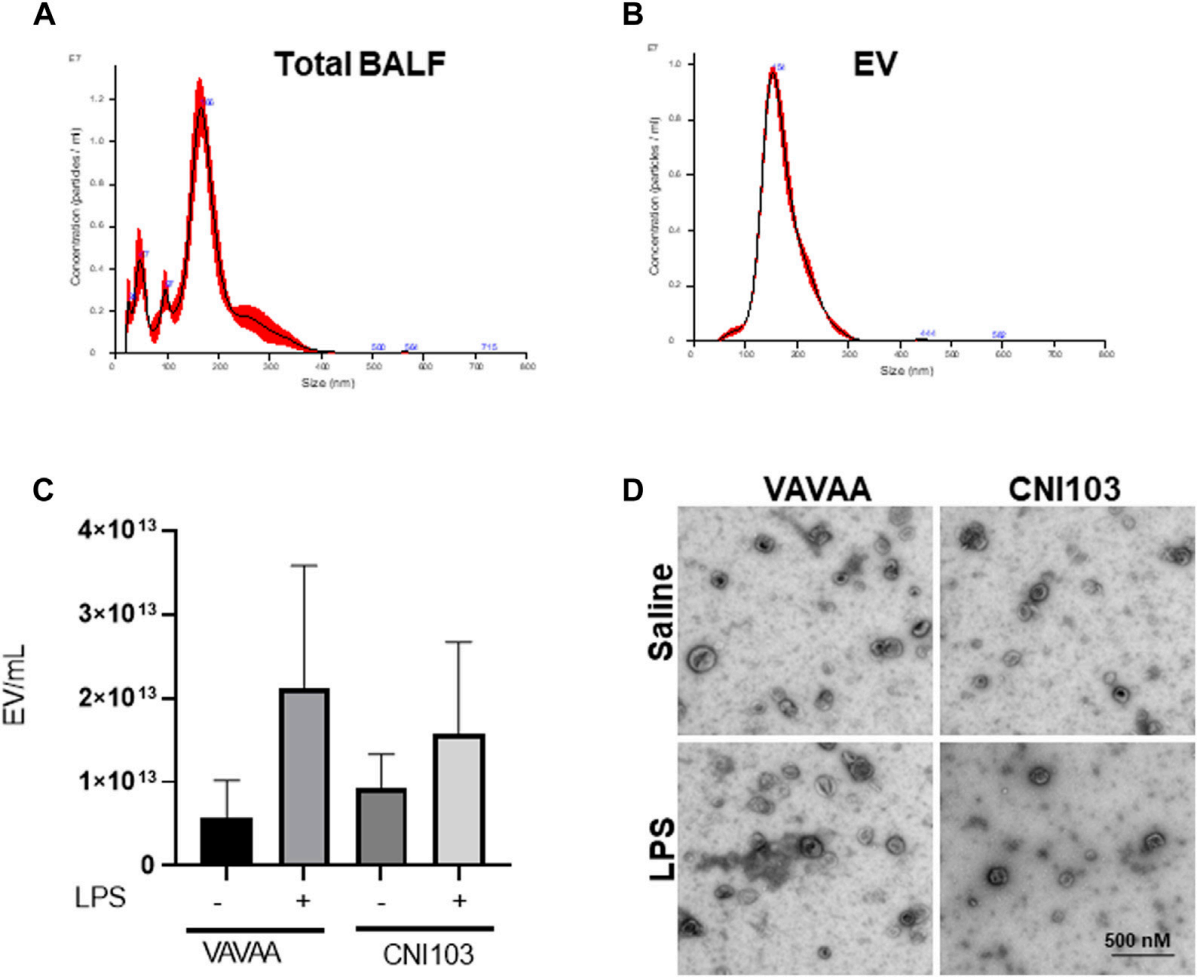
Calcineurin inhibitor CNI103 does not alter the release of EVs into the alveolar space in ALI mice. WT mice were pretreated with 4 mg/kg of control (VAVAA) or calcineurin inhibitor peptide (CNI103) by the intranasal route (i.n). Two hours after peptide delivery, the mice were further treated with saline or LPS (4 mg/kg in 25 µL saline i. n). Three days after the LPS challenge, mice were euthanized by ketamine and xylazine, and BALF was collected. EVs from the BALF of individual animals were isolated by ultracentrifugation and confirmed by nanoparticle tracking analysis for size. **(A)** Total BALF shows the size distribution of EVs ranging from 50 nm to 350 nm. **(B)** Purified population of EVs isolated by ultracentrifugation with a median size of 100–180 nm. **(C)** The numbers of EVs in the control and CNI103-treated mice are not significantly different. **(D)** Morphology of EVs captured by TEM.

To determine whether NFATc inhibition alters the release of EVs into the alveolar space, we analyzed BALF EVs from mice pretreated with CNI103 or a VAVAA peptide (Control) and then subjected to LPS-induced ALI or sham injury. All four groups of mice released EVs of varying sizes (50 nm–350 nm diameter) into BALF, analyzed by nanoparticle tracking (Figure 1A). Further centrifugation of the cell and MV-depleted BALF at 100,000 g and 4°C for 6 h yielded a homogeneous population of EVs with an average size of 150 nm (Figure 1B). Both the VAVAA- and CNI103-treated groups released a similar number of EVs into the alveolar space (Figure 1C). TEM images of the EVs confirmed the cup-shaped morphology and size of EVs to be within the 100–180 nm size range (Figure 1D).

**FIGURE 2:**
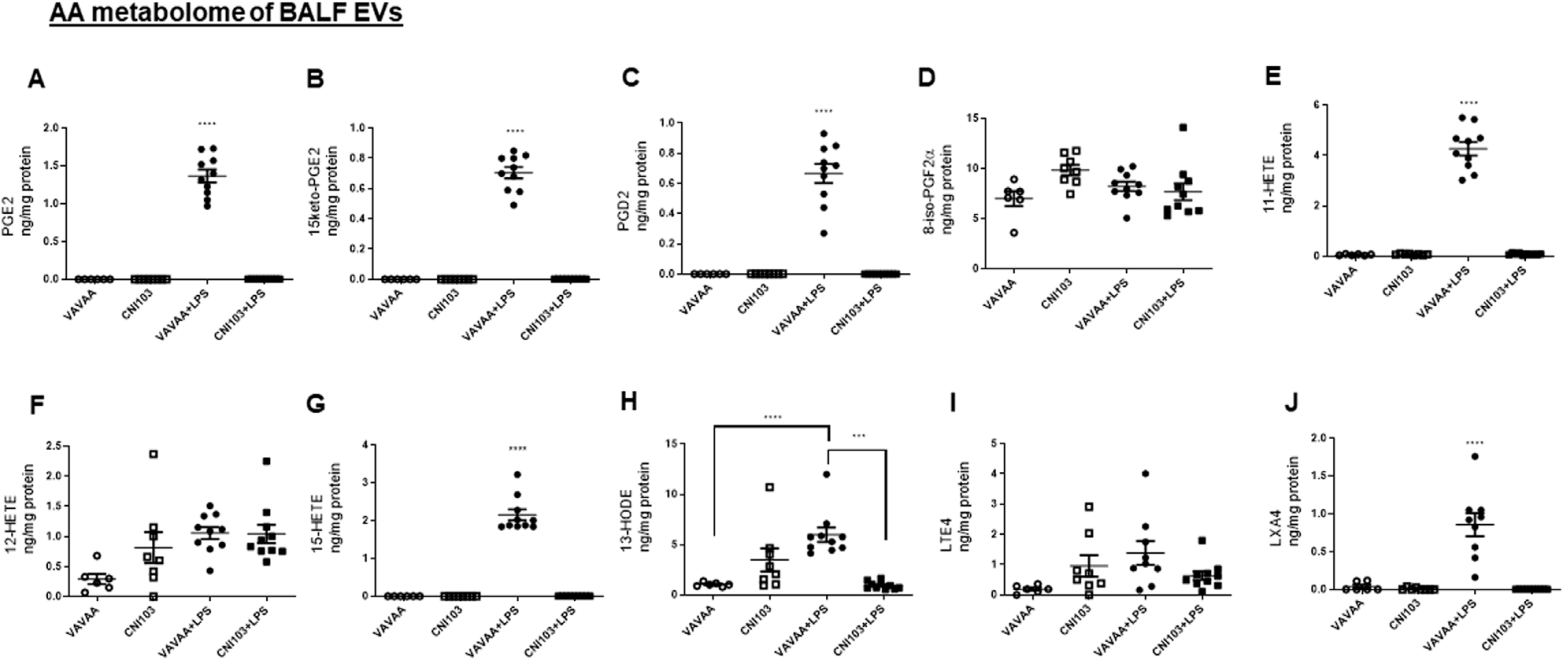
Calcineurin inhibitor CNI103 decreases the AA-derived eicosanoid content in BALF EVs. BALF EVs were isolated from control or CNI103-pretreated WT mice subjected to saline or LPS-induced ALI. Total lipids from EVs were extracted and analyzed by LC-MS using appropriate eicosanoid standards. Amount of COX metabolites in EVs: **(A)** PGE2, **(B)** 15keto-PGE2, **(C)** PGD2, **(D)** 8-iso-PGF2α and LOX metabolites, **(E)** 11-HETE, **(F)** 12-HETE, **(G)** 15-HETE, **(H)** 13-HODE, (**I)** LTE4, **(J)** LXA4, and **(K)** relative amount of eicosanoids in EVs from different experimental animals represented as a heat map. X-Undetected. n = 6–8 mice in the saline groups, n = 10 mice in the LPS groups. E ***p ≤ 0.001 or ****p ≤ 0.0001.

The lipid composition of the BALF EVs was analyzed by LC-MS/MS with different labeled eicosanoid standards. The EVs from mice treated with the control peptide or saline-treated mice had no detectable prostanoids (PGE2, 15keto-αPGE2, PGD2), hydroxyeicosatetrenoic acids (11-HETE and 15-HETE), or lipoxinA4 (LXA4) (Figures 2A–C,E,G,J). On the other hand, these EVs contained basal levels of 8-iso-PGF2α,12-HETE, 13-HODE, and LTE4 (Figures 2D,F,H,I). The LPS-induced ALI resulted in increased packaging of these lipid mediators into BALF EVs, correlating with the inflammatory state of lung injury. 8-iso PGF2α, which is a marker of oxidative stress and produced by the non-enzymatic peroxidation of AA in membrane phospholipids, was not elevated in the LPS-treated mice. Although LXA4 is known to function as a pro-resolution lipid mediator, it was packaged in the EVs along with pro-inflammatory mediators (Figure 2J). Notably, the BALF EVs from CNI103-treated mice subjected to lung injury had no detectable prostanoids, HETEs, HODE, or LXA4 (Figures 2A–C,E,G,H,J). However, the 8-isoPGF2α, 12-HETE, and LTE4 levels were similar between the control and CNI103-treated mice that were subjected to sham or LPS-induced lung injury (Figures 2D,F,I). Overall, the increase in the prostanoids, 11-HETE, 15-HETE, and LXA4 in response to lung injury was significantly decreased by CNI103 pretreatment, as illustrated in the heat map (Figure 2K).

### Pulmonary AA metabolism correlates with lipid mediators in BALF EVs

**FIGURE 3:**
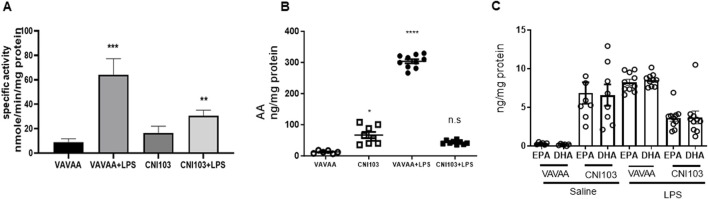
Catalytic activity of cPLA2 in lung tissue correlates with the amount of AA and eicosanoids in BALF EVs. WT mice were subjected to LPS-induced ALI as described in the *Methods* section. Right lung lobe homogenates from individual mice were prepared in a lysis buffer provided in a cPLA2 assay kit. **(A)** The specific activity of cPLA2 in lung homogenate was measured by hydrolysis of μmol of arachidonoyl thio-PC hydrolyzed per min per mg cellular protein. **(B, C)** The concentrations of free AA, DHA, and EPA are expressed as ng/mg EV protein. **n** = 6–8 mice in the saline groups, n = 10 mice in the LPS groups. *p ≤ 0.05 or **p ≤ 0.01 or ***p ≤ 0.001 or ****p ≤ 0.0001.

**FIGURE 4:**
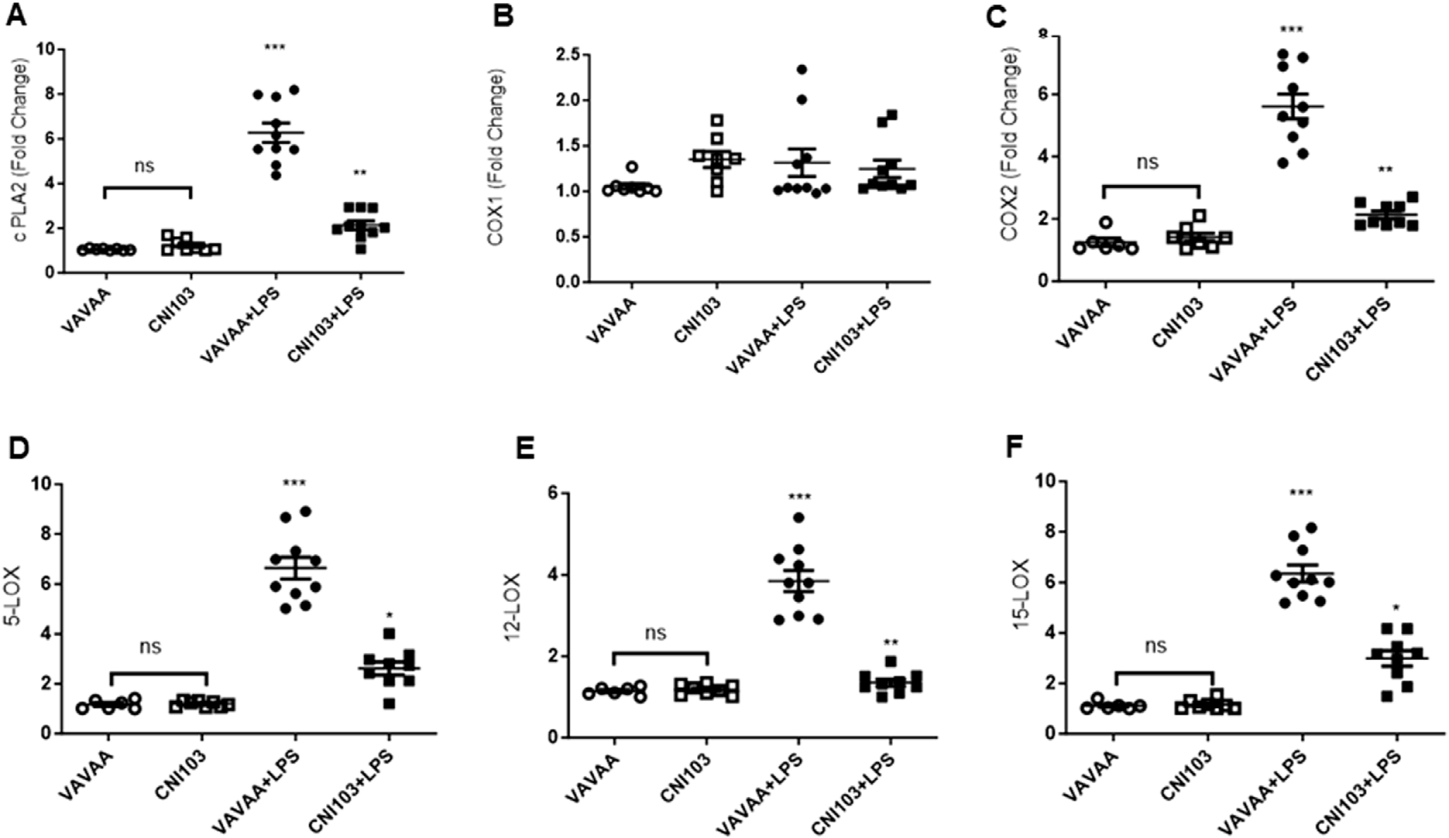
Calcineurin inhibitor CNI103 decreases the expression of AA-metabolic enzymes in lung tissue. WT mice were pretreated with 4 mg/kg of control (VAVAA) or calcineurin inhibitor peptide (CNI103) by the intranasal route (i.n). Two hours after peptide delivery, the mice were further subjected to a saline or LPS (4 mg/kg in 25 µL saline) challenge. Three days after the LPS challenge, mice were euthanized by ketamine and xylazine, and the lung tissue was perfused with 10 mL 1xPBS and snap-frozen in liquid nitrogen. Total RNA was isolated with a Direct-zol RNA Kit, and relative expression levels of **(A)** cPLA2, **(B)** COX-1, **(C)** COX-2, **(D)** 5-LOX, **(E)** 12-LOX, and **(F)** 15-LOX were measured and normalized with GAPDH levels.

**FIGURE 5:**
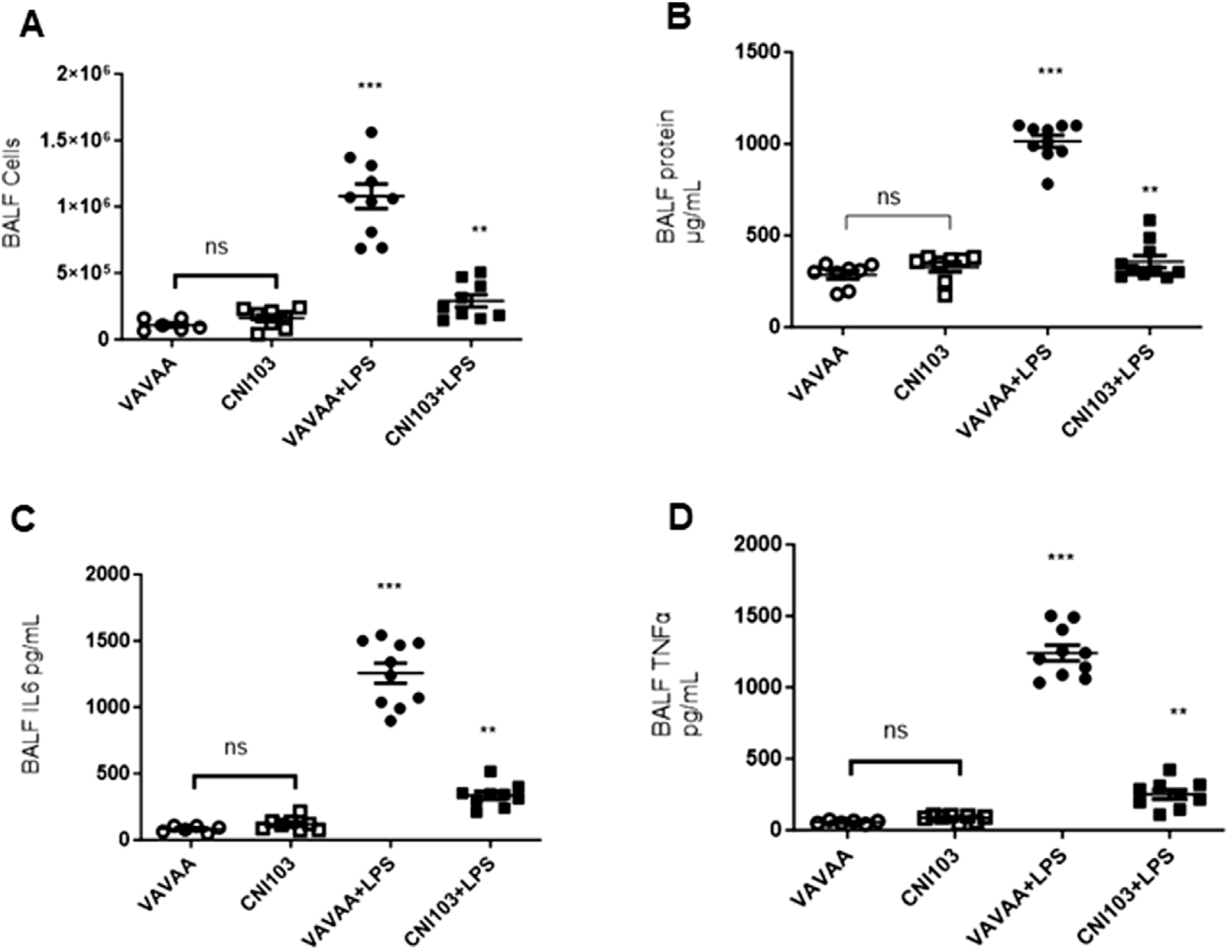
Calcineurin inhibitor CNI103 decreases the LPS-induced ALI-associated immune cell infiltration, protein extravasation, and cytokine storm in the alveolar space. WT mice were pretreated with 4 mg/kg of control (VAVAA) or calcineurin inhibitor peptide (CNI103) by the intranasal route (i.n). Two hours after peptide delivery, the mice were further subjected to a saline or LPS (4 mg/kg in 25 µL saline) challenge. Three days after the LPS challenge, mice were euthanized by ketamine and xylazine, and BALF was collected and analyzed. **(A)** Increase in immune cell infiltration into BALF. **(B)** Extravasated protein in cells and EV-depleted BALF. **(C, D)**. Relative amounts of IL6 and TNFα in cells and EV-depleted BALF. n = 6–8 mice in the saline groups, n = 10 mice in the LPS groups. **p ≤ 0.01 or ***p ≤ 0.001.

To determine whether the differences in lipid mediator content in the EVs were caused by increased substrate (AA) availability or increased catalytic proteins that generate these eicosanoids, we analyzed the lung tissue for cPLA2 activity, expression levels of cPLA2, and COX and LOX members. We found that both the specific activity and the expression of cPLA2 increased by 5–6-fold in the lung tissue of ALI mice as compared to the controls (Figure 3A. In contrast, the cPLA2 activity was significantly lower in CNI103-treated mice or equal to basal levels in the sham-injured mice. Furthermore, the amount of AA released in BALF EVs by the cPLA2 from membrane phospholipids is proportional to the enzymatic activity (Figure 3B). However, the DHA and EPA levels showed no correlation with cPLA2 catalytic activity. Unlike the release of AA from membrane phospholipids, the cellular content of DHA and EPA also depends on the conversion of α-linolenic acid to DHA and EPA by different chain elongase and desaturase enzymes, in addition to the availability of cellular PE and γ linoleic acid (Figure 3C). Expression levels of cPLA2 and downstream COX2, 5-LOX, 12-LOX, and 15-LOX, which generate different lipid mediators, increased upon LPS treatment, but this increase was suppressed by CNI103 treatment (4A, 4C–4F). On the other hand, COX-1 remained constitutively expressed during lung injury or upon CNI103 treatment (Figure 4B). As anticipated, there was increased infiltration of immune cells into the alveolar space in LPS-treated mice, which was abolished with the CNI103 treatment (Figure 5A. In all our previous studies, we measured cytokine storm and protein extravasation into cell-depleted BALF that still contained the MVs and EVs (Karpurapu et al., 2011; Karpurapu et al., 2018; Dougherty et al., 2020; Nie et al., 2021). Here, we analyzed EV-depleted BALF and found that the levels of IL6, TNFα, and extravasated protein were still high in LPS-treated mice. These inflammatory markers were diminished by CNI103 treatment (Figures 5B–D).

### EVs regulate PMVEC monolayer barrier functions

**FIGURE 6:**
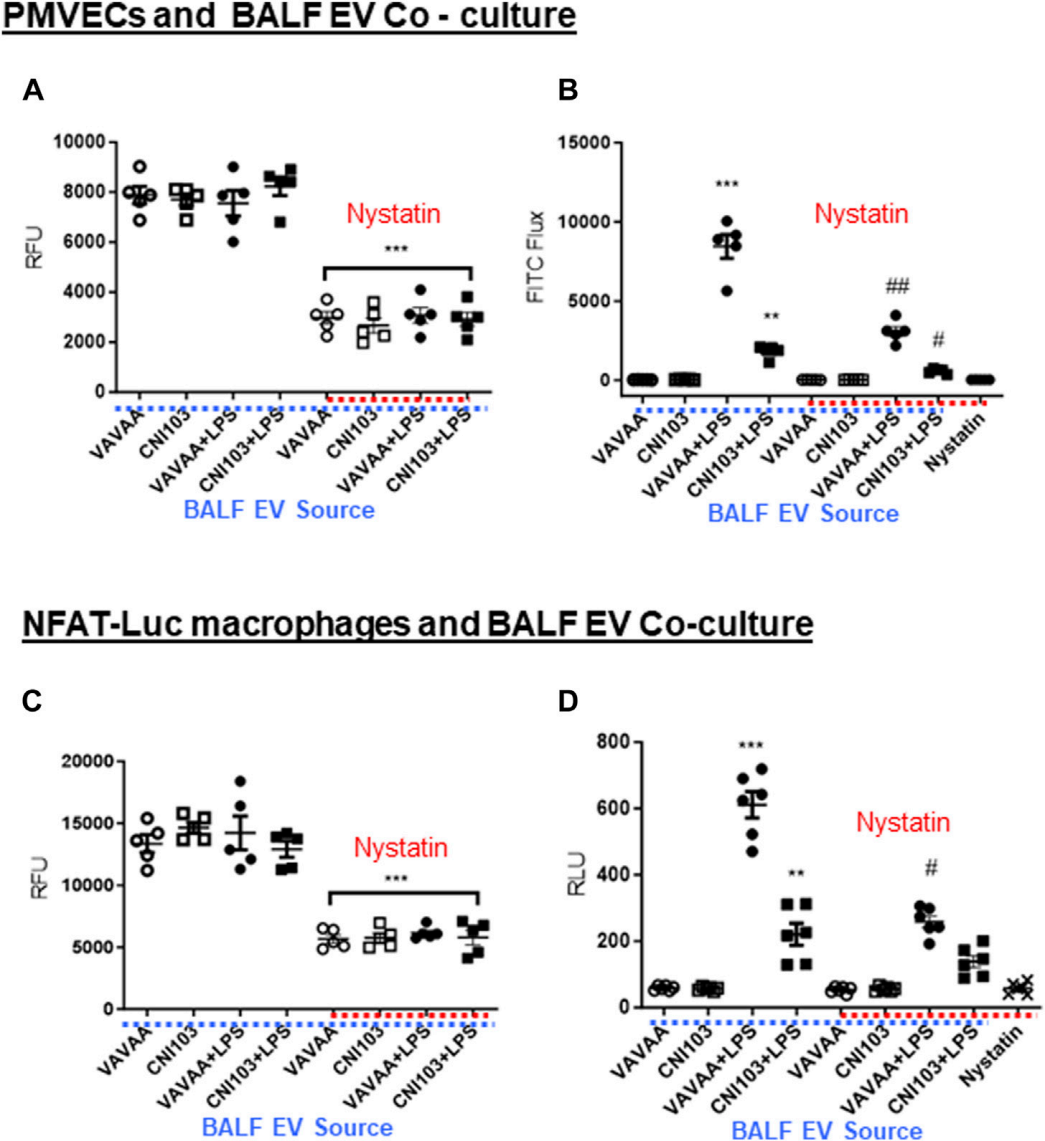
BALF EVs alter PMVEC monolayer barrier integrity and NFAT-luciferase activation. **(A)** Mouse PMVECs were co-cultured in the presence of PKH26-labeled EVs from control or CNI103 peptide-pretreated mice subjected to ALI or sham lung injury. In parallel, cells were pretreated (1 h) with DMSO (control) or nystatin (50 μg/mL) and co-cultured with EVs from different ALI mice for 16 h. EVs that were not internalized or adsorbed to cell membranes were removed by co-incubating with CD9 magnetic beads and washing twice with HBSS. EV uptake was measured as RFU. **(B)** Mouse PMVECs grown to 100% confluence on collagen-coated transwells were co-cultured for 24 h with EVs from control or CNI103 peptide-pretreated mice subjected to ALI or sham lung injury. In parallel, cells were pretreated (1 h) with DMSO (control) or nystatin (50 μg/mL) and co-cultured for 24 h with EVs from different ALI mice. A 0.1-mg/mL aliquot of FITC dextran was added to the transwells and incubated for an additional 1 h. After 1 h, the flux of FITC dextran into the medium in the bottom of the wells of the cell culture plate was measured at 492 nm/520 nm. **(C)** NFAT-luc macrophages were co-cultured for 24 h in the presence of PKH26-labeled EVs from control or CNI103 peptide-pretreated mice subjected to LPS-induced ALI or sham lung injury. In parallel, cells were pretreated (1 h) with DMSO (control) or nystatin (50 μg/mL) and co-cultured with EVs from different ALI mice for 16 h. EVs that were not internalized or adsorbed to cell membranes were removed with CD9 magnetic beads and washing twice with HBSS. EV uptake was measured as RFU. **(D)** Under similar experimental conditions, NFAT-luc macrophages were stimulated with unlabeled EVs from different ALI mice for 24 h. Cells were washed twice with HBSS and lysed, and relative luciferase activity was measured using the Renilla luciferin substrate. */#*p* ≤ 0.05 or **/##*p* ≤ 0.01 or ***p ≤ 0.001.

Lipid mediators play a pivotal role in the regulation of endothelial cell barrier function. We have determined the paracrine effect of EV-packaged lipid mediators on PMVEC monolayer barrier function in co-cultures. Prior to these studies, the uptake of PKH26-labeled BALF EVs by PMVECs was examined in co-cultures. PMVECs have internalized EVs from all four groups of mice with equal efficiency indicated by similar RFU units (Figure 6A). However, the EV uptake decreased by more than 50% in cells pretreated with nystatin, which is an EV uptake inhibitor. Co-culture of EVs from ALI mice disrupted the PMVEC barrier integrity. The flux of FITC through PMVEC monolayers was significantly higher in co-cultures of EVs from ALI mice than in sham injury mice (Figure 6B). Notably, FITC flux was significantly lower, comparable to the basal state in EVs from CNI103 and nystatin pretreatment, indicating a tighter monolayer barrier.

### EVs regulate macrophage function

The uptake of PKH26-labeled EVs from ALI and sham injury mice was similar in NFAT-luciferase reporter macrophage cells, which decreased by more than 50% by nystatin treatment (Figure 6C). EVs from ALI mice increased the NFAT-luciferase activity 8–10-fold compared to EVs from sham injury mice (Figure 6D). More importantly, the luciferase activity was significantly lower in cells co-cultured with EVs from CNI103-treated mice or EV uptake inhibitors.

## Discussion

Our prior studies have established that macrophage-PMVEC cross-talk plays a pivotal role in ALI pathophysiology (Karpurapu et al., 2018). We have identified that the calcineurin-regulated transcription factor NFATc3 plays intricate roles in the regulation of pulmonary macrophage function. NFATc3 regulates the release of chemokines and cytokines and the expression of toll-like receptor 5, iNOS, and kininogen 1 (Karpurapu et al., 2018). Furthermore, the inactivation of NFATc3 by CNI103 significantly decreased the intensity of LPS-induced ALI by downregulating the cytokine storm, neutrophilic influx, and pulmonary edema in mouse lungs (Dougherty et al., 2020). In the current study, we discovered a novel paracrine mechanism by which EVs released into the alveolar space regulate the barrier integrity of PMVECs. Studies from our group and others have linked pulmonary inflammation, alveolar epithelial cell, and microvascular dysfunction to the cytokine storm, oxidative stress, cell-free hemoglobin, and certain lipid mediators released during ALI (Matthay et al., 1984; Birukova et al., 2007; Shaver et al., 2016; Huppert et al., 2019; Bos and Ware, 2022). These factors collectively contribute to the cellular dysfunction and apoptosis of alveolar epithelial and microvascular endothelial cells. Lipid mediators, specifically eicosanoids, mediate various biological effects, including cell adhesion, endothelial cell activation, and the production of cytokines. In this context, the upstream cPLA2 is a key regulator of AA release and endothelial dysfunction in acute lung injury caused by both sterile and infectious insults (Cioccari et al., 2020; Htwe et al., 2021). More importantly, diverse lipid mediators, including prostaglandins, leukotrienes, phospholipids, sphingosine-1 phosphate, and oxidized lipids, were extensively researched for their regulatory role in the PMVEC barrier function (Jacobson and Garcia, 2007; Ke et al., 2017; Karki and Birukov, 2018; Fu et al., 2022). These lipid mediators alter diverse cell signaling pathways and can influence the actin cytoskeleton rearrangement in endothelial cells and the tight and gap junction proteins. Changes in the cytoskeleton are a key factor in the regulation of endothelial barrier function, as they affect the integrity of cell–cell junctions and overall vascular permeability.

Compared to cytokines, chemokines, and oxidant stress, EVs can exert a more pronounced paracrine effect. This is attributed to their structural integrity, ability to reach distant cells through circulation, and ability to transport biological cargo. The release of lipid bilayer-bound EVs appears to be a highly conserved physiological process among organisms from prokaryotes to humans (Yanez-Mo et al., 2015). More importantly, EVs have been shown to transport proteins, nucleic acids, and lipids to recipient cells. EV membranes are enriched in saturated lipids, offering stability to their cargo. Recent studies indicate that EVs released during ALI carry microRNAs that regulate inflammasome activation and the NF-κB pathway (e.g., miR-466, miR-223/142, miR-17, and miR-221 miR-92a-3p) (Lee et al., 2016; Chen et al., 2017; Lee et al., 2018). Similarly, MVs from mice subjected to LPS-induced ALI contained significant amounts of TNFα and lower amounts of IL-1β/IL-6 than healthy control mice (Soni et al., 2016; Soni et al., 2022). Furthermore, the culture of MLE-12 cells with the MVs from LPS-treated ALI mice induced the expression of epithelial intercellular adhesion molecule-1 (ICAM-1) and keratinocyte-derived cytokine (KC) release. These MVs induced neutrophilic infiltration when delivered into the tracheas of mice. In contrast, MVs released from pneumolysin-stimulated lung epithelial cells were shown to carry mitochondrial cargo and suppress neutrophil oxidative bursts (Letsiou et al., 2021). Our unpublished studies indicate that the MV BALF of LPS-treated mice transports a diverse array of lipid mediators. The loading of bioactive macromolecules into EVs appears to be a complex process that depends on the cell type, stimuli that activate signaling pathways, and endosomal sorting (Leidal and Debnath, 2020; Qiu et al., 2022).

To our knowledge, no published studies have investigated the “lipid constituents of EVs” from ALI patients or animal models. Furthermore, *ex vivo* lung perfusion studies with myeloid cell-derived EVs implicated them as mediators of acute lung injury that increased expression of receptors for advanced glycation end products (RAGE) by epithelial cells (Tan et al., 2023). In similar lines, using magnetic bead isolation techniques, we have detected an increased number of epithelial and neutrophil-derived EVs released into alveolar space during LPS-induced ALI (Nirujogi et al., 2022). In the current study, we have used the total pool of EVs from BALF, which represents the total lipidome from lung tissue, to determine their paracellular effect on PMVEC barrier function. We performed a high-throughput tandem LC-MS/MS analysis of BALF EVs from LPS-treated mice that revealed about 140 different lipid mediators originating from both ω-3 and ω-6 PUFA metabolism (Nirujogi et al., 2022). We have detected significant amounts of prostanoids (PGD2, PGE2, and PGF2α), HETEs (5-HETE, 12-HETE, and 15-HETE), HODEs (9-HODE and 13-HODE), and trace amounts of pro-resolution lipid mediators including LXA4, resolvin D5, and maresin in these EVs. This observation intrigued us, given the substantial number of studies implicating the role of various lipid mediators in the regulation of PMVEC barrier permeability. In the current manuscript, we have analyzed whether the protective effect of calcineurin inhibitor CNI103 is mediated by the EV lipid mediator content. We have observed that inhibition of NFATc with CNI103 significantly decreased the prostanoids, 11-HETE, 15-HETE, 13-HODE, and LXA4 in BALF EVs from LPS-treated mice. Notably, CNI103 decreased the catalytic activity and expression of cPLA2 that generate AA, the primary substrate for generating diverse eicosanoids. In addition, CNI103 also inhibited the expression levels of COX2, 5-LOX, and 15-LOX in lung tissue, which could be the plausible reason for lower amounts of prostanoids and HETEs in EVs. These results indicate a multi-level regulation of AA metabolism by the calcineurin–NFATc axis. In summary, we discovered a new role of the NFATc–calcineurin axis in regulating the lipid mediators in EVs by controlling the catalytic activities of AA-metabolic enzymes.

## Data Availability

The original contributions presented in the study are included in the article/Supplementary Materials; further inquiries can be directed to the corresponding authors.
